# Application of next generation sequencing (NGS) for descriptive analysis of 30 genomes of *Leishmania infantum* isolates in Middle-North Brazil

**DOI:** 10.1038/s41598-020-68953-9

**Published:** 2020-07-23

**Authors:** Kátia Silene Sousa Carvalho, Wilson José da Silva Júnior, Marcos da Silveira Regueira Neto, Vladimir Costa Silva, Sérgio de Sá Leitão Paiva Júnior, Valdir Queiroz Balbino, Dorcas Lamounier Costa, Carlos Henrique Nery Costa

**Affiliations:** 1 Laboratory of Leishmaniasis, Federal University of Piauí, Teresina, Brazil; 2 Laboratory of Bioinformatics and Evolutionary Biology, Federal University of Pernambuco, Recife, Brazil; 3 Center of Intelligence for Emerging and Neglected Tropical Injuries and Diseases, Teresina, Brazil; 4 Institute of Tropical Diseases, “Natan Portella”, Teresina, Brazil; 5 Department of Maternal and Childhood Health, Federal University of Piauí, Teresina, Brazil

**Keywords:** Computational biology and bioinformatics, Parasite genomics, Genomics

## Abstract

Visceral leishmaniasis (VL) is a life-threatening disease caused by the protozoa *Leishmania donovani* and *L. infantum*. Likely, *L. infantum* was introduced in the New World by the Iberic colonizers. Due to recent introduction, the genetic diversity is low. Access to genomic information through the sequencing of *Leishmania* isolates allows the characterization of populations through the identification and analysis of variations. Population structure information may reveal important data on disease dynamics. Aiming to describe the genetic diversity of *L. infantum* from the Middle-North, Brazil, next generation sequencing of 30 *Leishmania* isolates obtained in the city of Teresina, from where the disease dispersed, was performed. The variations were categorized accordingly to the genome region and impact and provided the basis for chromosomal ploidy and population structure analysis. The results showed low diversity between the isolates and the Iberic reference genome JPCM5. Most variations were seen in non-coding regions, with modifying impact. The ploidy number analysis showed aneuploid profile. The population structure analysis revealed the presence of two *L. infantum* populations identified in Teresina. Further population genetics studies with a larger number of isolates should be performed in order to identify the genetic background associated with virulence and parasite ecology.

## Introduction

Leishmaniasis is a group of diseases caused by different protozoan parasite of the genus *Leishmania,* transmitted by various sand fly species^1^. According to the World Health Organization (WHO), one billion people are living at risk of infection^2^. There are three major forms of the disease: cutaneous leishmaniasis, mucocutaneous leishmaniasis and visceral leishmaniasis (VL), or kala-azar (*calazar*, as it is broadly known in Portuguese), is the most severe and life-threatening presentation. Patients generally have prolonged fever, wasting, anemia and hepatosplenomegaly but may have a more severe clinical picture with jaundice, edema and dyspnea. Ten to twenty percent of patients die, usually with hemorrhage and bacterial infection. VL is a relevant opportunistic infection in the immunosuppressed living or visiting endemic areas. The clinical presentation depends on host as well and on parasite factors^3^.

VL is a neglected disease distributed in South and Central Asia, in the Middle East, East Africa, the Mediterranean and in Latin America, where over 90% of the cases are reported in Brazil, placing the country among those with the largest number of cases in the World^4^. VL dispersion and urbanization from the Northeast to the rest of the country and to the South Cone started in the 80’s due to unknown factors^5,6^. The two Middle-North states, Piauí and Maranhão, are among those with the largest number of cases. In the first, 245 VL cases were reported in 2017, with 86 from the state capital Teresina^7^.

*Leishmania* genomic data provided a plethora of information that revolutionized leishmaniasis research^8^, since it may identify the parasite influence on clinical presentation and epidemiological diversity of infectious diseases^9^. The complete genomes of *Leishmania* subgenus (*L. (L.) major*, *L .(L.) mexicana, L. (L.) tropica*, *L. (L.) amazonensis*, *L. (L.) donovani and L. (L.) infantum*^10–14^), the *Viannia* subgenus (*L. (V.) panamensis*, *L. (V.) braziliensis*, *L. (V.) guyanensis*, *L. (V.) naiffi*, *L. (V.) peruviana*, *L. (V.) lainsoni*^15–17^), as well as the *Sauroleishmania* subgenus (*L. (S.) tarentolae*, *L. (S.) adleri*^18,19^) and *Mundinia* subgenus (*L. (M) enriettii*, *L. (M.) macropodum*, *L. (M.) martiniquensis*^20^) are currently available*. L. infantum* genome was completely resequenced and assembled, showing 36 chromosomes with the total size of 32.134.935 base pairs. However, a single genome cannot estimate the genetic diversity of the species^21^. Therefore, it is of high interest to study the genomic architecture of specific parasite populations^9^.

Comparative genomic analysis is a powerful tool for discovering intrinsic genetic characteristics of a range of individuals belonging or not to the same species. Such characteristics may be associated with ecology, epidemiology, pathogenicity or virulence mechanisms of *Leishmania* spp.^22^. Genomic comparison between isolates of *L. donovani* and *L. major* identified genes involved in virulence and tissue tropism after infection in an animal model^23^ and in human disease^24^. Genomic sequencing also allowed the identification of different populations of *L. infantum*^25^. In addition, genomic variations such as aneuploidies (ploidy is the number of complete sets of chromosomes in a cell), single nucleotide polymorphisms (SNPs) and structural variants (SVs) like copy number variation (CNV), may affect the presence, dosage, and consequently expression of gene alleles related to virulence. SNPs, CNVs and aneuploidies have been suggested to be responsible for changes in virulence levels^26^.

Phylogenetic and population structure analysis have undergone significant increases due to the progressive advancement in sequencing technology. These events have subsidized exponential growth of genomic data, providing increasingly accurate and robust results. The present study aimed to describe the genetic diversity of 30 isolates of *L. infantum* isolated in Brazil, using a new generation sequencing approach. The representative isolate of species *L. infantum*, JPCM5 (isolated from a dog in Spain, in 1998), was used as a reference for genome assembly. The genomic analysis of the isolates first registered the number of variants (SNPs, insertions and deletions) present in each sample, and then categorized these variations as to genome location and impact. Population and phylogenetic structuring analysis were performed using SNP data, since they can be presented in binary form.

## Results

### Description of *L. infantum* isolates

All thirty isolates were obtained from the bone marrow of symptomatic humans diagnosed and treated in a reference hospital in Teresina, Brazil. The states Piauí and Maranhão, where the patients lived, compose the Middle-North, Brazil, and Teresina is situated right in the border between them. The farthest city from where patients lived is located at 460 km straight line distance from Teresina.

### Description of variants found in genomes of *L. infantum*

Table 1 shows the total number of SNPs, insertions, and deletions identified in the genomes of the 30 isolates of *L. infantum*. The number of SNPs ranged from 903 to 1,498 in isolates 3116 and 2145, respectively. SNPs were found in coding regions and were grouped into three categories regarding their effect on genomes: missense, nonsense and silent. In general, the number of SNPs in these categories is proportional to the total number of SNPs present in the isolate’s genome. Therefore, isolate 3116 presented the lowest number, while isolate 2145 presented the highest. The average value of the total amount of SNPs per isolate was calculated, as well as the three SNP categories and these values are shown in Table 1. Eleven isolates showed a below the average number of SNPs and the remaining 19 had an equal or above average total SNPs. This phenomenon was also noticed for the three categories of SNPs.

**Table 1:** Distribution and characterization of variants found in 30 isolates of *L. infantum* from the states Maranhão and Piauí, Brazil, based on the reference genome JPCM5.

Isolate	SNP	INS	DEL	Missense	Nonsense	Silent	Ratio
1213	997	1,487	1,597	213	2	111	1.92
1220	957	1,484	1,582	214	1	104	2.06
1255	1,237	1,155	1,046	305	4	142	2.15
1470	983	1,473	1,399	198	1	91	2.18
1661	1,172	1,087	1,024	264	3	119	2.22
1689	1,218	1,094	1,057	291	4	132	2.20
1798	1,290	1,169	1,096	296	3	153	1.93
1801	1,234	1,108	1,030	288	3	138	2.09
2008	1,192	1,130	1,026	282	3	135	2.09
2145	1,498	1,296	1,351	384	4	176	2.18
2492	1,192	1,117	1,054	291	4	138	2.11
2525	1,217	1,145	1,072	284	3	127	2.24
2527	1,273	1,190	1,074	302	3	147	2.05
2578	1,019	1,552	1,612	234	3	127	1.84
2765	1,232	1,043	1,007	295	4	122	2.42
2914	1,219	1,161	1,051	295	4	138	2.14
2959	1,205	1,104	997	296	3	128	2.31
3097	1,226	1,133	1,052	295	3	142	2.08
3113	1,300	1,221	1,122	297	4	140	2.12
3116	903	1,389	1,361	191	1	89	2.15
3130	1,243	1,193	1,048	297	3	139	2.14
3144	1,135	1,041	961	255	3	116	2.20
3148	1,247	1,247	1,078	269	4	122	2.20
3149	1,242	1,166	1,052	291	3	135	2.16
3151	1,266	1,270	1,148	292	4	141	2.07
3153	1,153	1,062	1,013	270	2	127	2.13
3167	1,261	1,140	1,059	306	3	140	2.19
3169	1,183	1,112	1,012	268	3	125	2.14
3170	1,186	1,018	1,006	293	3	123	2.38
3171	1,147	1,047	982	272	3	125	2.18
Average	1,188	1,194	1,132	278	3	130	2.14

All variants were categorized as single nucleotide polymorphism, insertion or deletion. The polymorphisms were classified as silent, missense or nonsense.

*SNP* single nucleotide polymorphism, *INS* insertion, *DEL* deletion, *Ratio* missense/silent.

When the number of insertions and deletions were evaluated, an opposite phenomenon to SNPs distribution was observed. The total number of insertions and deletions per isolate below the average was higher than the number above the average. Isolate 2578 stood out with the highest number of insertions and deletions, while isolates 3144 and 3170 showed the lowest numbers of deletions and insertions, respectively.

### Variant analysis for distribution in genome regions

**Figure 1:**
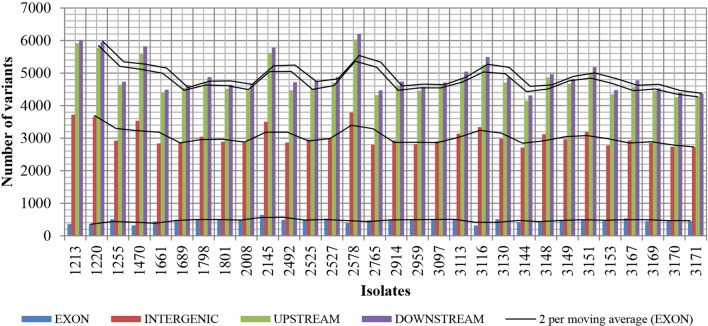
Graphical representation of the variants distribution according to genome regions (exon, upstream, downstream, intergenic). The SNPs, insertions and deletions were categorized according the region on the genome, considering different genes. A specific variant can be in different region for different genes. Most of the variations are in downstream and upstream (non-coding) regions of the genes.

Given the number of mutations found in the genomes, the richness of these modifications was evaluated in the introns, e.g. the non-coding regions of the genome (intergenic, downstream and upstream to genes) and exons (coding regions) (Fig. 1). When the number of mutations was summarized, the most was observed to be situated in the downstream and upstream regions to genes, followed by the intergenic and exonic regions. Notably, although the number of mutations was similar within the four regions, some isolates, stood out by presenting a greater number of mutations than the others (isolate 2578) or by having the lowest (isolated 3144), both for the intergenic, upstream and downstream regions. Regarding the exonic regions, isolates 2145 and 3116 presented the highest and lowest number of mutations, respectively.

### Variant analysis for impact in the genome

**Figure 2:**
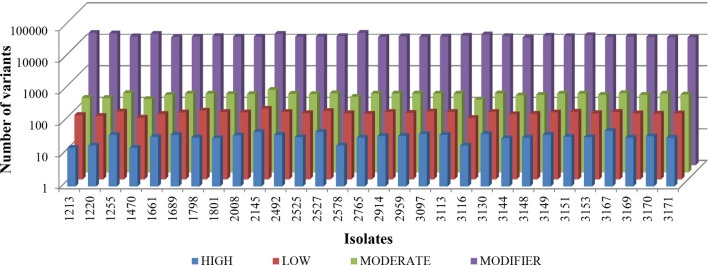
Distribution of variants according to the impact (high, low, moderate, modifier) on the genome. Most of the SNPs, insertions and deletions showed to have the modifier impact category for the genome. The least amount of variation is of high impact for the genome.

A survey of variations in the genomes of 30 *L. infantum* isolates was performed and they were classified according to the impact on their respective regions of the genome (Fig. 2)*.* Impact categories were classified in the following way: high impact mutations as those affecting splice sites, start and stop codons; moderate impact as non-synonymous variations; low-impact as synonymous variations in coding regions and start and stop codons; and as modifier variations in non-coding region (upstream, downstream, intergenic and UTR regions). The number of modifying variations was significantly larger than those with high, low and moderate impact. The isolate 2145 had the largest number of high, low and moderate impact categories of SNPs taken altogether (n = 640). On the opposite side, the isolates 1470 and 3116 had the smallest. Relative to the impact modifier category, the isolate 2578 had the largest number of SNPs (16,023) and the isolate 3144 the smallest (11,180).

### Variant distribution by chromosome

**Figure 3:**
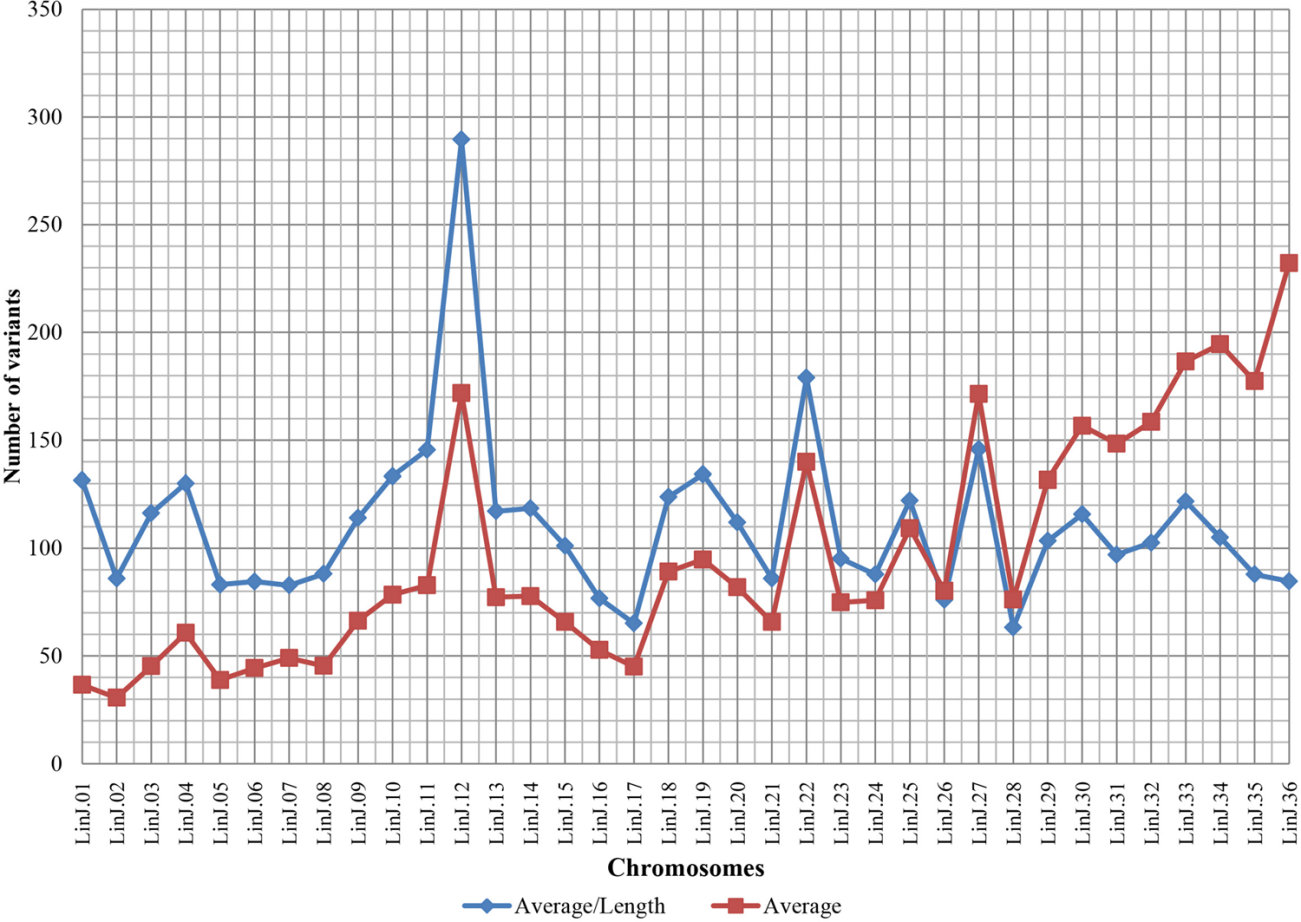
Distribution of variants by chromosome. The SNPs, insertions and deletions were classified by chromosome. The chromosomes 12, 22, 27 and 36 have the largest amount of variation in relation to JPCM5. Considering the length, the chromosome 36 showed to have little variation.

Figure 3 shows the number of variants in each chromosome. Chromosomes 12, 22, 27 and 29 to 36, had the highest crude number of variants. After normalizing the number of variants by the size of the chromosomes, only chromosomes 12, 22 and 27 remained as those with larger number and chromosome 12 stood out as one with the highest number of variants.

### SNPs density

**Figure 4:**
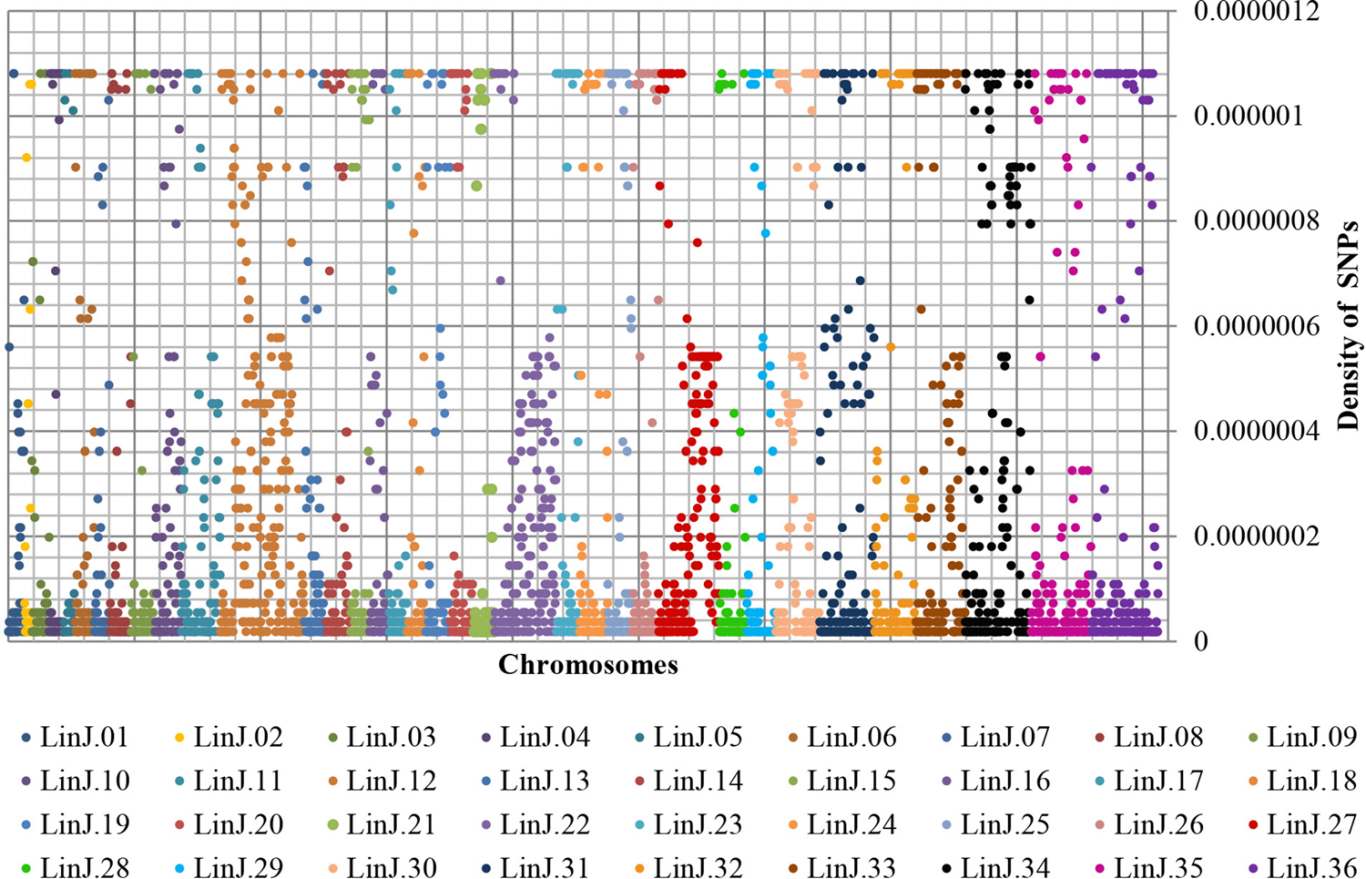
Density of SNPs by chromosome of the 30 genomes of *L. infantum* isolates. The SNPs were classified by chromosomes and the density was calculated. The low density can be observed mainly in chromosomes 12, 22 and 27.

Figure 4 shows that a significant number of SNPs are not evenly distributed across the genomes. The phenomenon is seen in all chromosomes but more markedly in chromosomes 12, 22 and 27.

### Chromosome copy number variation

**Figure 5:**
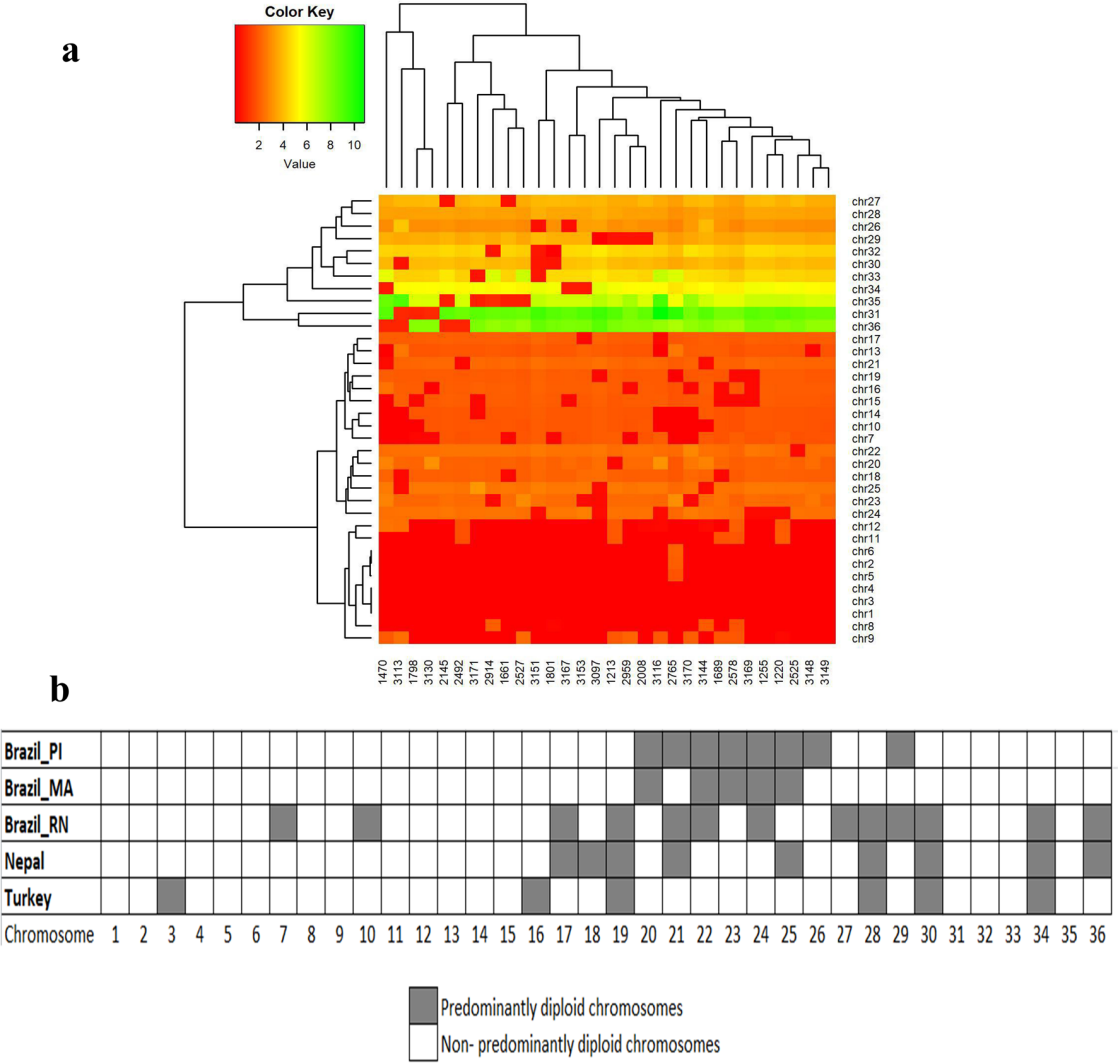
Evaluation of chromosomal copy number per isolate. (**a**) The heatmap shows the estimated copy number of the 36 chromosomes (y-axis) in the isolates (x-axis). Above the heatmap denote three groups of isolates formed by a hierarchical clustering analysis of the somy values. (**b**) Distribution of disomic chromosomes amongst five groups of *L. infantum* and *L. donovani* isolates*.* Three groups are from different states of Brazil: Piauí, Maranhão and Rio Grande do Norte^25^. The others two groups are from Turkey^27^ and Nepal^13^. The isolates from Piauí and Maranhão showed similar pattern but different from the others isolates regarding the disomic chromosomes. PI: Piauí; MA: Maranhão; RN: Rio Grande do Norte.

The ploidy number for every isolates was estimated by comparison with the reference *L. infantum* genome JPCM5^14^. According to the results (Fig. 5), the disomic condition was observed for most chromosomes (25/36). The remaining 11 chromosomes (26, 27, 28, 29, 30, 31, 32, 33, 34, 35 and 36) showed some degree of aneuploidy. Chromosomes 31 and 36 are tetrasomic, with the exception of isolates 1,470, 3,113, 1,798, 3,130, 2,145, 2,492, which presented disomy for the same two chromosomes. The karyotypes of chromosomes 26, 27, 28, 29, 30, 32, 33, 34 showed one or two extra chromosomes in most of the analyzed isolates.

A cluster analysis based on the values of ploidy was performed with the 30 isolates and, as observed in Fig. 5, ploidy analysis grouped the isolates into three clusters as indicated by the dendrogram at the heatmap top. This dendrogram grouped the disomic isolates in chromosome 31. The dendrogram at the left side of the heatmap grouped the 36 *L. infantum* chromosomes in three groups, according to the degree of ploidy. In this dendrogram it is possible to visualize that the shorter chromosomes are more stable in relation to aneuploidy. When five groups of *L. infantum*^28^ and *L. donovani*^13^ isolates from different origins were compared in relation to ploidy, it was possible observe a distinct pattern of aneuploidy in chromosomes of isolates from Piauí and Maranhão.

### Genetic structuring and population phylogeny

**Figure 6:**
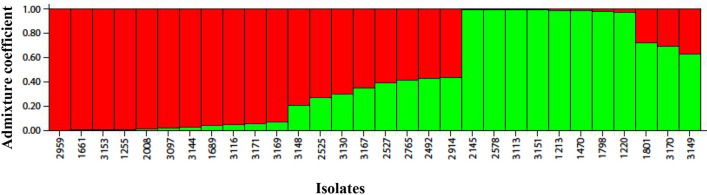
Bar graph generated by STRUCTURE software inferring the genetic structure of the 30 isolates of *Leishmania infantum*. It was revealed the presence of two populations, red and green, in the sample of this study.

**Figure 7:**
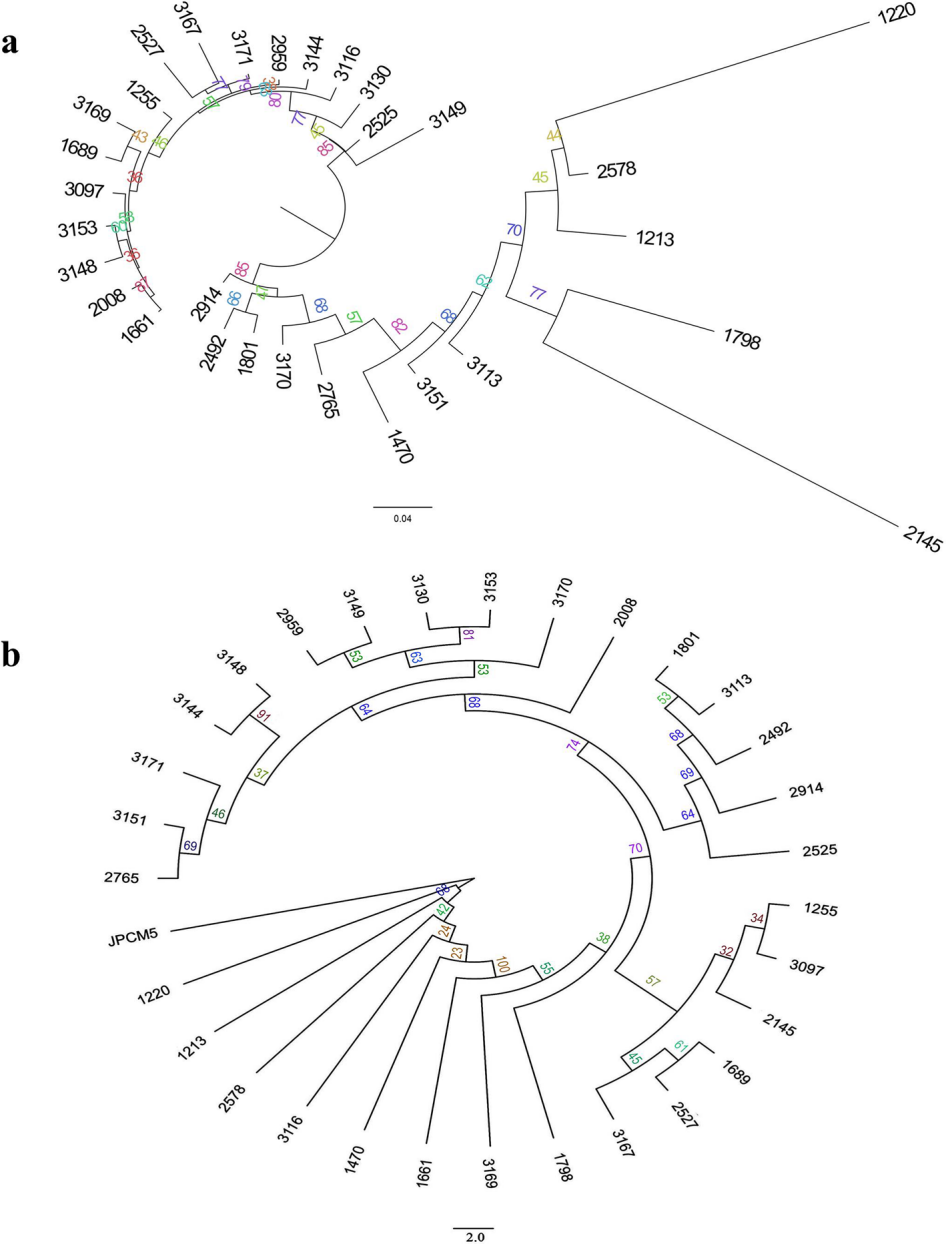
Maximum likelihood tree obtained with the (**a**) MK + FQ + R3 and (**b**) MK + FQ + ASC + R3 models chosen according to the Baysian Criterion Inference with 100,000 bootstrap, showing the results of the analysis of all SNPs of *L. infantum* isolates. (**a**) The tree topology consistently distinguished two monophyletic groups, clade 1 and clade 2, both with 85% bootstrap values. (**b**) The tree topology shows the genetic proximity with the reference JPCM5.

Data on the presence or absence of SNPs from each isolate was used in the genetic structuring analysis using the STRUCTURE software (Fig. 6). This algorithm identifies genetically distinct populations and estimates the coefficient of association of individuals in each probabilistic population. The peak in ΔK represents the most likely number of populations and subpopulations^28^. Genetic structure analysis indicated that the isolates are divided into two groups, as supported by the value of K = 2. The tree generated with the data from all SNPs, through maximum likelihood analysis (Fig. 7), divided the isolates of *L. infantum* in two clades, corroborating the genetic structuring analysis. The geographical distribution of the patients is depicted in the Supplementary Table S1 and Supplementary Fig. S1.

## Discussion

This article provides relevant whole genome information about the largest number of isolates of *L. infantum* from Brazil ever analyzed, all collected from symptomatic humans with VL. In addition to the analyses of the genetic structure of populations, phylogeny and ploidy analysis have already been shown in a previous study with *L. infantum.* This work brings meaningful information about the amount of variation by isolate and by chromosome, including analysis in their type and impact for the genome.

Complete genome sequencing is the most appropriate approach to identify species and isolates, as well as their variants, especially when closely related individuals are analyzed^29^. Genetic characterization of *L. infantum* isolates was accessed through quantitative and qualitative analysis of the variants, which allowed the verification of the most frequent variation types and their effects, and the genetic structure of the isolates.

The isolates showed a number of similar variant types in reference to canine Iberic isolate JPCM5. These numbers allow inferring a relative genetic proximity between isolates of *L. infantum* from the Old and New World^30^ and a recent migration of New World populations that created a genetic drift with a bottleneck effect. The small amount of variation was even lower than that observed in the SNPs count among distinct *L. donovani* isolates (over 3,000 SNPs) and *L. infantum* (1,250 SNPs), the last one from northeastern populations of *L. infantum* from Brazil^13,25^. Moreover, this conclusion took in account the resequenced reference genome the JPCM5 isolate with the complete assembly of the 36 contigs (chromosomes)^14^ in contrast to the two previous assemblies that used as reference genome the data from the first assembly of JPCM5, with incomplete genome regions (gaps) and 37 scaffolds^11,14^. Additionally, the low level of genetic differentiation of this set of parasites is showed by the presence of only 2 to 3% of SNPs in the coding regions, albeit the complete *Leishmania* genome has 48% in the coding region^10^. The proportion observed now was indeed lower than that presented in other studies, comparing with other isolates from *L. infantum* (7–8%)^25^ and *L. donovani* (4–5%^31^ and 17%^13^) suggesting the most recent introduction of *L. infantum* in Middle-North Brazil. *L. donovani* seems to have dispersed even earlier as it has a higher accumulation of variation^32,33^.

Among the mutations in coding regions, silent or synonymous mutations are expected to appear in greater numbers, which did not happen in this study. However, Zackay et al.^34^ found no significant difference between the percentages of detected synonymous, non-synonymous and nonsense mutations in *L. donovani* from Ethiopia. Synonymous variants do not alter the primary structure of polypeptide, but may have negative effects on the stability and structure of mRNA and proteins, and may contribute to the complexity of the infectious diseases, being interesting targets for the identification of genetic factors associated with virulence^35^. For example, in *L. infantum*, nine silent SNPs were detected in the malic enzyme gene (class of enzymes that catalyze the reduction of NADP + and needed to maintain intracellular redox homeostasis of the parasite), one of which distinguishes strains from the same zymodemo^36,37^. Also, several genes associated with drug resistance development were shown to contain non-synonymous SNPs or nonsense mutations^34^. Additionally mutations in exonic regions have been demonstrated to help differentiate isolates from a population and knowing about their impact on different *Leishmania* isolates’s pathogenicity^31,37^. Finally, the presence of non-synonymous SNPs in genes located in conserved regions, important for parasite metabolism^31^ and associated with increased parasite load^30^, classify these genes as candidates to detect differences in virulence from isolates and species. To characterize the genes where these variations are and then to identify virulence factors are tasks for next steps of our studies.

In all isolates used in this study, most variants showed modifying impact. The modifying impact, usually exhibited by mutations present in non-coding regions of the genome, such as downstream, upstream, intergenic, 3′UTR and 5′UTR regions of the genes, have a difficult effect to predict or to highlight^38^ . However, it can result in functional consequences for organisms, such as translation and regulation in response to environmental changes^39^. Intergenic regions, in particular, increase the availability of essential genomic sites to respond to growth conditions modifications through gene amplification^40^ but, as seen by Figueiredo de Sá et al.^29^ most SNPs were present in downstream and upstream regions of genes.

The greatest amount of variation found in this study was also noticed in some of the same chromosomes that showed the greatest nucleotide diversity in the previous study with isolates of *L. infantum*^25^, such as chromosome 12 and the largest chromosomes, 33, 34, 35 and 36. Among the chromosomes that stand out for having the highest number of mutations is also chromosome 22, where the A2 family genes are located, and which are important genes for the survival and visceralization of *L. donovani*^40^. Interestingly, normalization by the length of the chromosome showed the disproportionality between the number of variants and the size of chromosome 36 since it is not the one with largest number of variants despite having the largest number of genes.

*Leishmania* spp. genome is characterized by the presence of long clusters of polycistronic genes, with almost complete absence of introns and by a high gene density^39^. However, *L. braziliensis* SNPs were homogeneous, characterizing low density^29^ and in the present study the SNPs were not uniformly distributes.

Substantial variations in chromosomal copy number of *L. infantum, L. major, L. donovani* have been identified^12,25,31,41^. This variation might be related to the differences in the pathology and ecology of the isolates within the same species, since the increased copy number of chromosomes allows the amplification and overexpression of genes that play a role in pathogenesis^31^ and adaptation to drug pressures in *L. donovani*^42,43^ and to the environment. Previous study of the karyotype of *Leishmania* spp. revealed it has 36 chromosomes^44^, albeit the presence of aneuploid chromosomes^45^, as it can be also be observed in this article. Aneuploidy, a feature which showed being correlated whit SNP variation^44^, is well tolerated by *Leishmania* and allows rapid generation of parasite diversity, proving to be one of the main adaptation strategies^45^. In *Leishmania*, a cell population may appear in various ploidy states (monosomic, disomic or trisomic), generating a heterogeneity between strains called “mosaic aneuploidy”^16^. This was revealed not only by ploidy instability among the isolates of this study but also by the variable chromosome content of the cultivated population (presence of intermediate values for chromosome copy number^25^). However, without cloning the parasitic cells, this observation needs further checking. This phenomenon of aneuploidy may be caused by the high rate of asymmetric chromosome division during nuclear division, which leads to its transmission over the following generations^44^ or variations in culture methods, culture time, growth conditions used in different laboratories^12^. Gene dosage tolerance due to 'mosaicism' can be explained by post-transcriptional and translational regulation in *Leishmania*, given that there is little control over the level of transcription^46^.

Chromosome 31 and its homologous are known as supernumerary chromosomes in different species and lineages of the genus *Leishmania*^12,16,25^. Their ontological analysis has shown that this chromosome is enriched in genes involved in iron metabolism and other molecular functions^16^ essential for parasite survival. Although it is unclear why this chromosome is specifically driven by the largest numerical changes, one of the hypotheses is a low percentage of coding region, which would need to increase its copy number to meet the cellular need for the transcripts produced by its genic content^25^. In this study, interestingly, chromosome 31 showed a disomic pattern in some isolates. Therefore, further studies are necessary to explain gene expression in this chromosome in order to clarify this unconventional phenomenon.

The cluster analysis of the isolates for ploidy showed the formation of three groups, when one of them grouped the isolates with disomic chromosome 31. However, increased ploidy is generally inversely related to chromosome size and the shorter chromosomes the more stable they are regarding to aneuploidy^47^ and this is not related to disease status, isolation time, host, and SNP differences^25^.

The population structure of *L. infantum* might help to elucidate epidemiological aspects, such as the spread of parasites in endemic areas and the origin of VL outbreaks. Previous studies of genetic structure based on 14 microsatellite markers had already allocated isolates from Middle-North Brazil within the same population. This population was the largest in most collection hubs that comprised all regions of Brazil^48^. However, due to the greater discriminatory power of genomic sequencing and using a larger sample size that increased the discriminatory power, this study revealed the presence of two populations in the region, although isolated only from humans. The consistency of this result is illustrated by the generation of the phylogenetic tree with two distinct branches. Such groups were checked if a geographical pattern could be noticed as it happens in other regions of the world^49^. If fact, one of the two populations was more concentrated in a circle with a ray of 200 km around the city of Teresina, which interestingly, was the first larger city in Brazil where urban epidemics waves started, in 1981^5^. Additional analyses are on the way to clarify this phenomenon.

The evolution of parasites is influenced by the genetic structure of the population, that can be stimulated by geographical barriers, parasite distribution, vector and reservoirs’ biology, strongly interfering with the homogeneous dissemination of genotypes^28,50^. The sample size used in this study increase the reliability in the result, but in order to access more data on the evolution of populations, including data about drug resistance^44^ it is suggested a deeper analysis on the genetics of the populations, with larger sample size. Given that the clinical and epidemiological spectrum of VL in Brazil is wide, with different degrees of virulence recorded these results open perspectives for future work to try to find a genetic pattern of the parasite that would explain the difference in host pathogenesis and transmission ecology.

## Methods

### Sample preparation

Thirty *L. infantum* isolates from patients treated at the reference hospital Natan Portella Tropical Diseases Institute in Teresina. The parasites frozen and stored at − 196 °C were used. The patients lived in different cities of Piauí and Maranhão states (Supplementary Table S1).

After thawing, the parasites were grown in NNN medium (Novy-McNeal-Nicolle) and Schneider’s medium (Insect Medium, Schneider, Sigma, St. Louis, USA), supplemented with fetal bovine serum (10%), sterile urine (2%), gentamicin (80 mg), 100 U/mL penicillin and 100 µg/mL streptomycin (Pen/Strep Gibco, Grand Island, NY, USA). After seven days, observing the viability of the parasites, a passage in 10 mL of supplemented Schneider’s medium was performed. Upon reaching the exponential phase, in about 5 days the tubes containing the parasites were centrifuged at 3,000 rpm for 10 min at 4 °C and the pellet was washed three times with physiological solution (0.9% NaCl). After the last wash, the parasites were resuspended in 200 µL of 0.9% NaCl and proceeded to DNA extraction.

### DNA extraction and sequencing

200 µL of the solution containing *Leishmania* was used for DNA extraction using Genomic DNA Pure Link Mini Kit (INVITROGEN) according to the manufacturer's instructions. DNA was quantified by using QUBIT 2.0 Fluorometer and NANODROP 2000/2000c spectrophotometer, and the purity of the samples was also verified by observing the 260 nm/280 nm ratio. The quality of the extracted DNA was certified by 1% agarose gel electrophoresis. Isolated DNA was sequenced by MACROGEN, INC., Soeul, through the ILLUMINA Next Generation Sequencing (NGS) platform using the HiSeq2500 Sequencer and the TruSeq DNA PCR-Free Library Prep Kit.

### Read quality assessment and coverage calculation

The reads were initially evaluated by the FastQC v0.11.7^51^ program, where it was possible to verify the quality and the presence of adapters. With the evaluated reads, the sequencing coverage calculation was based on the Lander/Waterman formula and was calculated using the Eq. (1)^52^:

1 $${\text{C}} = {\text{L*B*N}}/{\text{G}}$$

where C is the genome coverage, L is the average length of the reads, B is the type of library used for sequencing (being 1 and 2, single-end and paired-end, respectively) N is the total number of reads (per library) and G is the total size of the haploid genome.

### Mapping of *reads* in the reference genome

The reads were aligned in the reference genome of *L. infantum* JPCM5^14^ using BWA v14r0.7.17-r1188 (BWA-MEM)^53^ with the default parameters. Samtools v1.7^54^ was used to convert SAM files to BAM, sort, remove duplicates and index data. Then using PicardTools v2.20.8^55^ all reads were assigned to a new reads group, and finally GATK v3.7.0^56^ was used to call variants that generated the VCFs files containing all variant information by genome.

### Variant and SNP filtering and data transformation

For variant filtering, bcftools^57^ with parameters “QUAL > 30 && MQ > 30” was used to generate the filtered VCFs. For the annotation of variants regarding region and impact, SnpEff v4.0^38^ was used and the database was created for the reference genome used in the assemblies. SNPs were obtained by removing INDELS from the VCFs files. VCFs with only SNPs were concatenated to obtain a matrix created by VCFTools^58^ with parameters—012, where it was agreed that 0 is the absence of SNP, 1 is the heterozygous presence and 2 is the homozygous presence. All the isolates were genotyped based on presence or absence of the SNPs.

### Chromosome number analysis

To calculate the number of chromosome copies, the methodology of Zhang et al.^31^ was used. For this purpose the bamCoverage tool was implemented in the deepTools^59^ program package to estimate the depth of each chromosome, and then normalize the data using the RPKM method. The median depth of each chromosome (*d*_*chr*_*)* was obtained and then calculate the total depth of all chromosomes using Eq. (2),

2 $$\left( {d_{T} \, = \,median \, [d_{chr1} \ldots d_{chr36} ]} \right).$$

Finally, the chromosomal copy number was then defined by using Eq. (3)

3 $$S_{chr} \, = \,d_{chr} /\left( {d_{T} /2} \right).$$

These steps were done in Excel 2010^60^ assuming previously that in general all chromosomes are diploid.

### Phylogeny and population structure

The matrix generated by VCFTools was transformed into a nexus file to mount a maximum likelihood analysis by the IQtree^61^ software, which chose the evolutionary model MK + FQ + R3 defined by the Bayesian Criterion Inference. To check the relationship beetween these isolates and the reference JPCM5, the same process was performed by using the evolutionary model MK + FQ + ASC + R3. The consistency of the branches was verified using 100,000 boostraps. For the structuring of the population, STRUCTURE^62^ was used. The interactions were made with 20,000 warming interactions, followed by 200,000 Markov and Monte Carlo chain generations, adjusted from 1 to 10 for the “K” population. The values of ΔK were obtained in order to accurate the number of populations "K".

### Ethical approval

The isolates were prepared for genomic analysis, particularly for high throughput sequencing with the aim of identifying virulence factors. This publication is the first part of the whole study, approved by the Research Ethics Committee of the Federal University of Piauí (approval ID number 0116/2005). All methods were performed according to the approved guidelines and regulations. A written informed consent was obtained from all study participants or their legal guardians.

## Supplementary information

Supplementary Information

## Data Availability

The datasets generated and analyzed during the current study are available in the NCBI repository, https://www.ncbi.nlm.nih.gov/bioproject/589999.
