# Supplementary material for: Application of next generation sequencing (NGS) for descriptive analysis of 30 genomes of Leishmania infantum isolates in Middle-North Brazil
Source: Sci Rep. 2020 Jul 23;10:12321. doi: 10.1038/s41598-020-68953-9 (PMC7378178; doi:10.1038/s41598-020-68953-9)

Application of *Next Generation Sequencing* (NGS) for descriptive analysis of 30 genomes of *Leishmania infantum* isolates in Middle-North Brazil

Kátia Silene Sousa Carvalho[1], Wilson José da Silva Júnior[2], Marcos da Silveira Regueira Neto[2], Vladimir Costa Silva[1,3,4], Sérgio de Sá Leitão Paiva Júnior[2],Valdir Queiroz Balbino[2], Dorcas Lamounier Costa[3,4,5], Carlos Henrique Nery Costa[1,3,4]*

[1] Laboratory of Leishmaniasis, Federal University of Piauí

[2] Laboratory of Bioinformatics and Evolutionary Biology, Federal University of Pernambuco

[3] Center of Intelligence for Emerging and Neglected Tropical Injuries and Diseases

[4] Institute for Tropical Medicine "Natan Portella"

[5] Department of Maternal and Childhood Health, Federal University of Piauí.

*Corresponding author: chncosta@gmail.com

Supplementary Material

Supplementary Table S1. Description of the sequenced isolates of *L. infantum* by origin by State, municipality, geographical coordinates and distance to Teresina

ID patiente	State	City	Geographical coordinate	Distance to Teresina in km (straight line)
1213	PI	Teresina	-5.04S -42.76W	0
1220	MA	Timon	-5.10S -42.83W	9.87
1255	MA	Timon	-5.10S -42.83W	9.87
1470	MA	Timon	-5.10S -42.83W	9.87
1661	PI	Floriano	-6.77S -43.02W	191.71
1689	MA	Aldeias Altas	-4.62S -43.46W	90.60
1798	PI	Piracuruca	-3.93S -41.70W	171.15
1801	MA	Codó	-4.45S -43.89W	140.22
2008	PI	Queimada Nova	-8.58S -41.41W	413.77
2145	PI	Floriano	-6.77S -43.02W	191.71
2492	MA	Codó	-4.45S -43.89W	140.22
2525	MA	Timon	-5.10S -42.83W	9.87
2527	PI	Elesbão Veloso	-6.20S -42.13W	141.56
2578	PI	Teresina	-5.04S -42.76W	0
2765	PI	Teresina	-5.04S -42.76W	0
2914	MA	Codó	-4.45S -43.89W	140.22
2959	PI	Colônia do Gurguéia	-8.18S -43.79W	363.35
3097	PI	São João do Piauí	-8.35S -42.25W	370.96
3113	MA	Codó	-4.45S -43.89W	140.22
3116	MA	Esperantinópolis	-4.87S -44.69W	213.71
3130	PI	Teresina	-5.04S -42.76W	0
3144	PI	Teresina	-5.04S -42.76W	0
3148	MA	Pedreiras	-4.57S -44.60W	201.29
3149	MA	Timon	-5.10S -42.83W	9.87
3151	PI	Teresina	-5.04S -42.76W	0
3153	PI	Aroazes	-6.11S -41.78W	160.90
3167	PI	Barras	-4.24S -42.29W	103.84
3169	PI	Bonfim do Piauí	-9.18S -42.88W	459.55
3170	MA	Capinzal do Norte	-4.72S -44.32W	176.59
3171	PI	Teresina	-5.10S -42.83W	0

PI: Piauí; MA: Maranhão.

Supplementary Figure S1. Overview of origin of *L. infantum* isolates. The map shows the localization of patients' municipality in Piauí and Maranhão states and the circle with their 200 km distance to Teresina, which shows the distribution of populations regarding this city. Each point is an isolate and their colors represent a population. The Rio Grande do Norte state from where the first Brazilian isolates with whole genomes sequenced is shown. The map was generated by ArcGIS 10.7 (https://www.esri.com/es-es/arcgis/about-arcgis/overview).

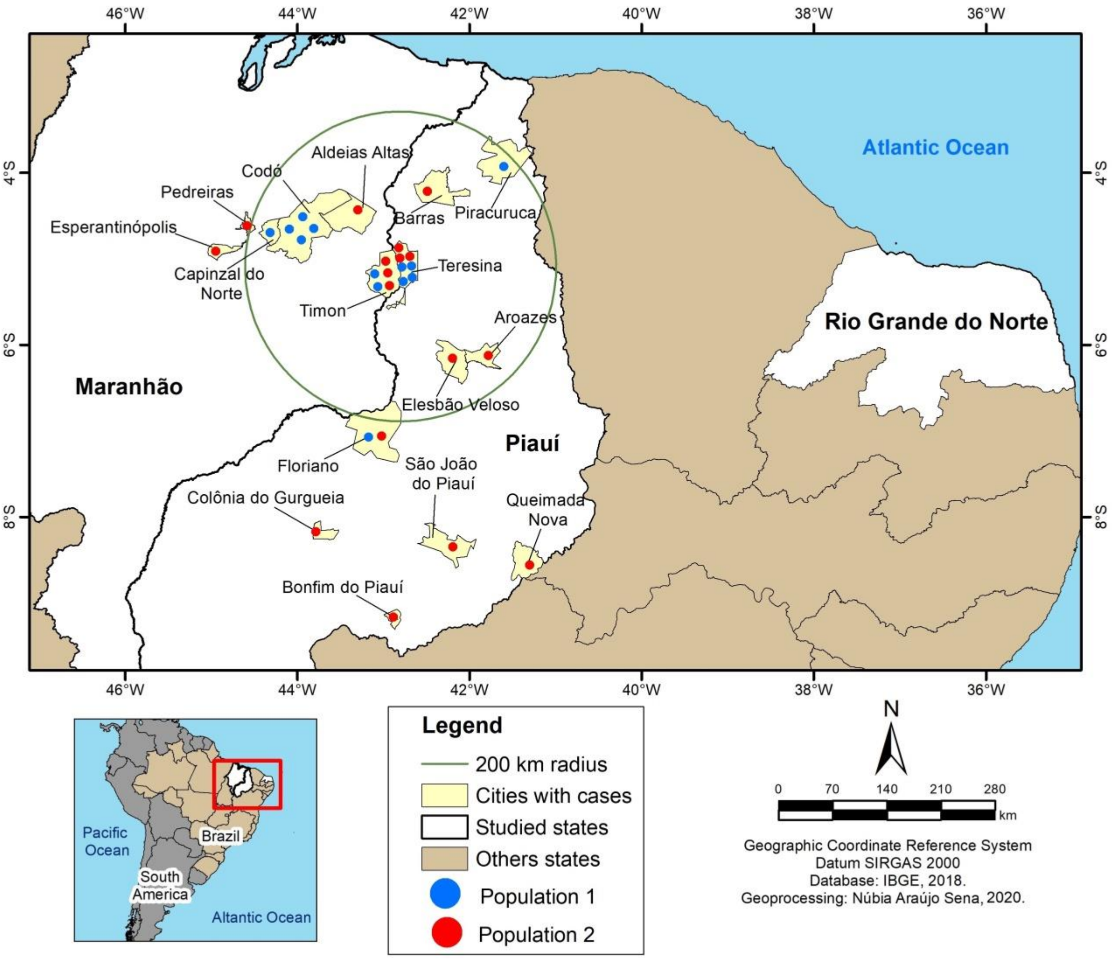

Supplement: Supplementary file 1 — Supplementary Information [file 41598_2020_68953_MOESM1_ESM.pdf]
